# Expression of human dopamine receptor in potato (*Solanum tuberosum*) results in altered tuber carbon metabolism

**DOI:** 10.1186/1471-2229-5-1

**Published:** 2005-02-09

**Authors:** Aleksandra Skirycz, Anna Świędrych, Jan Szopa

**Affiliations:** 1 Institute of Biochemistry and Molecular Biology, University of Wrocław, Przybyszewskiego Street 63/77, 51 – 148 Wrocław, Poland; 2 Max-Planck-Institute of Molecular Plant Physiology, Am Mühlenberg 1, D-14476 Golm, Germany; 3 Department of Plant Physiology University of Szczecin, Wąska Street 13, 71–415 Szczecin, Poland

## Abstract

**Background:**

Even though the catecholamines (dopamine, norepinephrine and epinephrine) have been detected in plants their role is poorly documented. Correlations between norepinephrine, soluble sugars and starch concentration have been recently reported for potato plants over-expressing tyrosine decarboxylase, the enzyme mediating the first step of catecholamine synthesis. More recently norepinephrine level was shown to significantly increase after osmotic stress, abscisic acid treatment and wounding. Therefore, it is possible that catecholamines might play a role in plant stress responses by modulating primary carbon metabolism, possibly by a mechanism similar to that in animal cells. Since to date no catecholamine receptor has been identified in plants we transformed potato plants with a cDNA encoding human dopamine receptor (HD1).

**Results:**

Tuber analysis of transgenic plants revealed changes in the activities of key enzymes mediating sucrose to starch conversion (ADP-glucose phosphorylase and sucrose synthase) and sucrose synthesis (sucrose phosphate synthase) leading to altered content of both soluble sugars and starch. Surprisingly the catecholamine level measured in transgenic plants was significantly increased; the reason for this is as yet unknown. However the presence of the receptor affected a broader range of enzyme activities than those affected by the massive accumulation of norepinephrine reported for plants over-expressing tyrosine decarboxylase. Therefore, it is suggested that the presence of the exogenous receptor activates catecholamine cAMP signalling in plants.

**Conclusions:**

Our data support the possible involvement of catecholamines in regulating plant carbon metabolism *via *cAMP signalling pathway.

## Background

The catecholamines (dopamine, norepinephrine and epinephrine) are a group of biogenic amines possessing a substituted 3, 4-dihydroxy phenyl ring that are widespread in the animal kingdom; but they have also been detected in plants [1,2]. The role of catecholamines in plants is poorly documented, but it is clear that they are involved in many aspects of growth and development. They were proposed as precursors for various alkaloids [3,4] and to be associated with processes such as ethylene production, nitrogen fixation, defence against herbivores, flowering, prevention of 3-indole acetic acid (IAA) oxidation and gibberellin signalling [5,6]. Analogous with animal cells in which catecholamines stimulate glycogen mobilization a similar role for catecholamines in the regulation of plant carbohydrate metabolism was suggested. Transgenic plants over-expressing tyrosine decarboxylase, which controls an important step of catecholamine synthesis, were characterized by highly increased concentrations of norepinephrine and soluble sugars, whereas starch level was dramatically decreased. Observed changes indicated a positive correlation of norepinephrine with soluble sugars and a negative correlation with starch [7]. The physiological action of catecholamines in animal cells is mediated by their interaction with G-protein coupled receptors that stimulate or inhibit the enzyme adenylyl cyclase (AC). In most animal cells cyclic AMP (cAMP) exerts its effect by activating cAMP dependent, serine-threonine protein kinase (PKA). Recently strong evidence for the occurrence and function of cAMP in higher plants has emerged [8]. It was demonstrated that cAMP levels in tobacco bright yellow 2 (TBY-2) cells are tightly connected to cell cycle progression [9]. The involvement of cAMP in gibberellin and ABA action has also been suggested [10,11]. Molecular evidence has shown the existence of plant protein kinases containing a high degree of sequence homology with PKA [12]. Moreover, molecular techniques led to the identification of cAMP response element-binding proteins (CREBs) [13], cyclic nucleotide-gated cation channels [14] and cAMP binding enzymes [15]. These data strongly indicate the involvement of catecholamines in regulating plant carbohydrate metabolism, possibly by a mechanism similar to that in animal cells. However, this suggestion is limited by the fact that to date no catecholamine receptor has been identified in plants. In the present study we characterize potato plants transformed with a cDNA encoding human dopamine receptor (HD1). The receptor is a rhodopsin-like integral membrane protein of 446 amino acids, seven transmembrane domains and molecular mass of 49 kDa. Our analysis revealed a regulatory effect of HD1 on carbohydrate metabolism including changes in key enzyme activities.

## Results

### Transgenic plant selection

**Figure 1:**
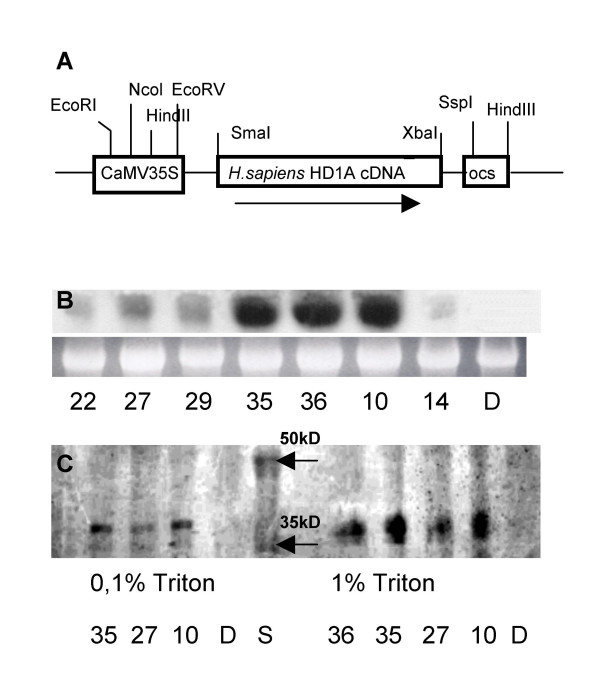
**Selection of transgenic HD1 plants**. Potato plant transformed with pHD1-BinAR construct carrying a cDNA for human dopamine receptor under the control of the CaMV 35S promoter (A) were selected by northern blot analysis (B). Lines showing the highest expression were further submitted to western blot analysis. For this purpose proteins were extracted with 0.1% or 1% Triton (C) and 50 μg of protein extract was run on 12% SDS polyacrylamide gel. D refers to control plants, numbers to different transgenic lines and S to Full Range Rainbow Molecular Weight Marker RPN 800 (Amersham Bioscience).

*Solanum tuberosum *plants transformed with pHD1-BinAR, a plasmid carrying a cDNA for the human dopamine receptor under the control of the CaMV 35S promoter (Figure 1A), were pre-selected by means of PCR with the primers for neomycin phosphotransferase (kanamycin) gene and then selected by northern blot analysis with a HD1 specific cDNA as a probe (Figure 1B). Four transgenic lines showing the highest mRNA expression of the expected length (1300 bp) were chosen for further analysis by western blot (HD1.10, HD1.27, HD1.35 and HD1.36). Using commercially available monoclonal rabbit IgG anti-HD1 antibody a ~37 kDa protein band was detected in transgenic plants. It was absent from control plants (Figure 1C). Surprisingly the protein was ~10 kDa smaller than expected which may suggest posttranslational modification. Careful inspection of the cDNA sequence revealed the presence of two translational signals (232 bp and 313 bp) that would result in 40 kDa and 37 kDa proteins, respectively. However, as the translational machinery is very similar in plants and animals we suggest that the short form of receptor resulted from proteolytic action rather than de novo synthesis. It should be pointed out that a stronger signal for HD1 expression was accompanied by a stronger protein signal (lines HD1.10; HD1.35; HD1.36). Conversely in plants with weak HD1 expression, the protein signal was comparatively weak (line HD1.27). HD1 extraction with 1% Triton was much more efficient than extraction with 0.1%Triton in agreement with the expected membrane localization of the HD1 protein.

### Phenotype analysis

Tubers of HD1 plants grown in a field were harvested after four months and analyzed. All examined transgenic lines produced more tubers per plant. The yield was not significantly changed since increase in tuber number was accompanied by decrease in tuber weight (Table 1). There were no obvious morphological differences between aerial parts of wild type Desiree and HD1 plants.

### Catecholamine level

In order to develop an easy and reliable assay for the quantitative and qualitative determination of catecholamines in plants, the suitability of gas chromatography coupled to a quadruple mass spectrometer (GCMS) was recently investigated. A sensitive GCMS method based on the analysis of the trimethylsilylated catecholamine derivatives was developed to monitor the presence and concentration of these compounds and related metabolites. Based on the retention times and the mass spectra of standards the presence of dopamine, norepinephrine and a new compound normetanephrine in potato leaves and tubers was clearly detected [2].

**Figure 2:**
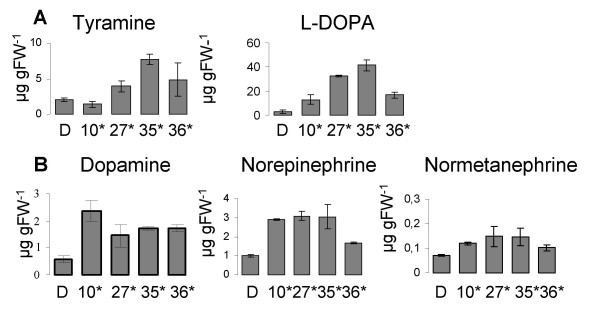
**Catechoalmines analysis**. GC-MS analysis of HD1 plants revealed changes in catecholamine levels. Concentration of catecholamine's precursors, tyramine and L-DOPA, is elevated as well. Results are mean ± SE of four independent measurements on three individual plants per line. *Asterisks *(*) indicate values that are significantly different from the wild type plants.

In contrast to the previous studies performed on plants over-expressing tyrosine decarboxylase [7], which controls an important step of catecholamine synthesis, the goal of our work was to stimulate an alternative signalling pathway by introducing human dopamine receptor. Surprisingly the expression of dopamine receptor resulted in a more than two-fold increase of dopamine, norepinephrine and normetanephrine in all transgenic lines examined (Figure 2B). This increase of catecholamines content was accompanied by significant increase of tyramine and L-DOPA, which are direct precursors of dopamine (Figure 2A). Despite changes in concentration of catecholamines and their precursors, the level of tyrosine, which serves as a precursor for tyramine and L-DOPA, was not altered (data not shown).

### Determination of carbohydrate content in tubers of transgenic plants

**Figure 3:**
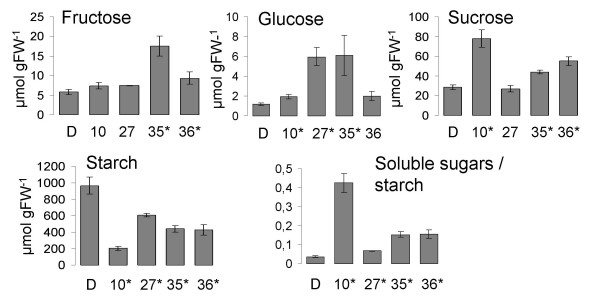
**Sugars analysis**. Tubers were harvested in September after 3 months of growth and the glucose, sucrose, fructose and starch contents were determined. Data represents the mean ± SE of determination on three individual plants per line. *Asterisks *(*) indicate values that are significantly different from the wild type plants.

Following our recent finding that the action of dopamine and norepinephrine in potato is on starch mobilization, we decided to analyze transgenic tubers expressing dopamine receptor for soluble sugars and starch content. All transgenic plants showed decreased starch content, with levels that ranged from 20 to 60% percent of the wild type. This was accompanied by a significant increase in soluble sugar concentration ranging from 2.7 to 1.15 fold in comparison to Desiree (Figure 3). Concentrations of starch and soluble sugars were highly correlated. The calculated correlation coefficients between catecholamines content and the levels of glucose, sucrose, fructose and starch were 0.38, 0.69, 0.38 and -0.95, respectively.

Changes in carbohydrate are most likely responsible for the altered phenotype of transgenic HD1 tubers. Reduced tuber mass can be explained by decreased starch content whereas increased tuber number by the increase of soluble sugar concentration.

### Sucrose – starch metabolism

Under normal growth conditions the major flux in potato tuber carbon metabolism is the conversion of sucrose through hexose phosphates to starch [16].

Since HD1 plants were characterized by changed concentrations of both soluble sugars and starch we measured the activities of enzymes involved in this pathway. Sucrose transported from leaves is symplastically unloaded from the phloem and degraded by sucrose synthase (SuSy). ADP-glucose phosphorylase (AGPase) converts glucose-1-phosphate (Glu-1-P) into ADP-glucose, an immediate precursor of starch. Both SuSy and AGPase are considered as key enzymes for starch synthesis [17].

**Figure 4:**
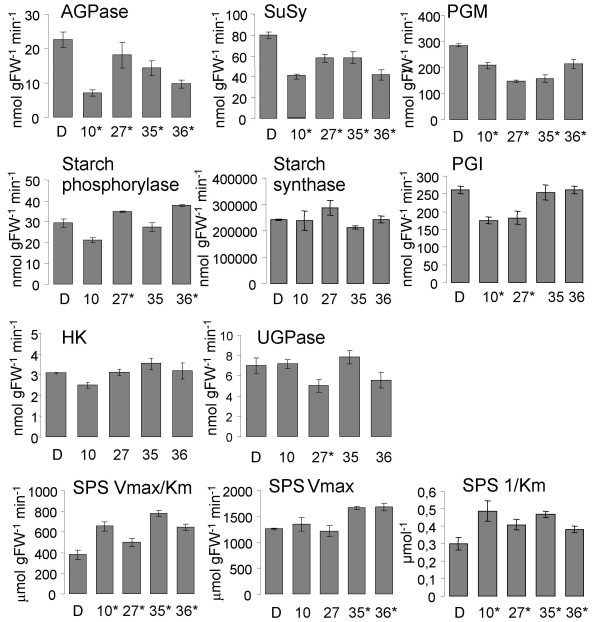
**Enzyme activities**. The activity of enzymes involved in sucrose and starch metabolism in tubers of control (D) and HD1 plants. Enzyme activities were measured in the same tuber's samples as the one used for carbohydrate, catecholamine and metabolite analysis. Data represent the mean ± SE of determination on three individual plants per line. *Asterisks *(*) indicate values that are significantly different from the wild type plants. SPS – sucrose phosphate synthase; UGPase – UDPglucose pyrophosphorylase; AGPase – ADPglucose pyrophosphorylase; SuSy – sucrose synthase, PGM – phosphoglucomutase; PGI – phosphoglucoisomerase; SS – starch synthase

Activities of AGPase and SuSy were significantly decreased in HD1 plants to 56% and 68% of the wild type level, respectively (Figure 4). In agreement with their roles in starch synthesis, and their proposed coordinated regulation, activities of both enzymes and starch content were all significantly correlated (cor >0.9).

Phosphoglucomutase (PGM) catalyzes the conversion of glucose-1-phosphate to glucose-6-phosphate. Tubers are characterized by the presence of cytosolic and plastidial isoforms of phosphoglucomutase. Repression of either of them results in plants with decreased starch levels pointing out the importance of the enzyme for starch accumulation [18,19]. The activity of PGM was significantly decreased in all transgenic lines, most likely contributing to the reduction in starch synthesis (Figure 4). Activities of other enzymes involved in sucrose-starch conversions (hexokinase, UGPase and starch synthase) were not changed significantly (Figure 4). In most of the transgenic lines inhibition of starch synthesis was accompanied by increased hexose-6-phosphates (Table 2).

To establish whether enhanced starch mobilization also contributed to the observed decreases in starch content we measured the activity of starch phosphorylase. In two out of the four examined transgenic lines the activity of starch phosphorylase was significantly increased, further contributing to decreased starch content of HD1 plants (Figure 4)

Moreover HD1 expression led to activation of sucrose phosphate synthase (SPS), responsible for sucrose production. Maximum SPS activity (measured wih saturating substrates, Vmax) only changed in two lines, whilst activity of the enzyme measured in the assay that contained limiting substrate concentration (Vmax/Km) and as a consequence 1/Km increased in all the lines.

1/Km, correlated well with the sucrose content of transgenic tubers (cor -0.81) (Figure 4).

### Glycolysis/TCA cycle

**Figure 5:**
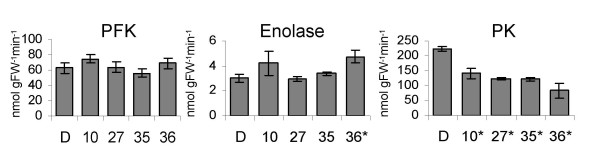
**Glycolytic enzyme activities**. Activities of the enzymes involved glycolysis in tubers of HD1 plants. Enzyme activities were measured in the same tuber's samples as the one used for carbohydrate, catecholamine and metabolite analysis. Data represent the mean ± SE of determination on three individual plants per line. *Asterisks *(*) indicate values that are significantly different from the wild type plants. PFK – phosphophructokinase; PK – pyruvate kinase.

The high concentrations of glucose and glc-6-P measured in the HD1 plants indicated changes in the glycolytic pathway. However, activities of glycolytic enzymes (hexokinase, phosphofructokinase and enolase) were not changed. The only exception was pyruvate kinase, which showed a significant decrease of activity in all transgenic lines (Figure 5). To investigate if this reduction of activity led to changes in carbon metabolism *via *the TCA cycle we measured the content of TCA intermediates. In all transgenic lines citric acid, isocitric acid and malate were significantly reduced, while fumarate showed a significant increase (Table 2).

## Discussion

In contrast to the vast knowledge concerning the role and action of catecholamines in mammals, very little is known about the physiological significance of catecholamines in plants. Since most of the components of animal catecholamine signaling pathway have been also identified in plants (G-proteins, cAMP, PKA homologs) the involvement of catecholamines in plant signalling pathways is possible. Recently, the analysis of transgenic plants over-expressing tyrosine decarboxylase, which accumulate a high quantity of catecholamines, suggested a possible signalling effect on plant primary metabolism. The increase of catecholamines resulted in decreased starch concentration but increased soluble sugars [7].

**Figure 6:**
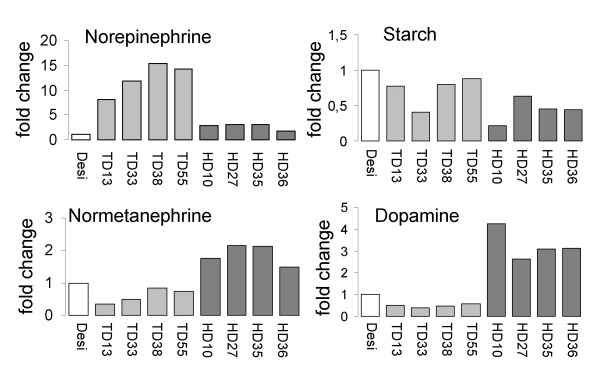
Comparison of starch and catecholamine content measured in tubers from HD1 and TD plants.

The only component of mammalian catecholamine signaling pathway that to date has not been identified in plants is the catecholamine receptor. We transformed potato plants with a cDNA encoding human dopamine receptor (HD1) in order to analyze whether the presence of a receptor affects the endogenous catecholamine action. Western blot analysis showed that the protein was produced in transgenic plants and biochemical analysis of transgenic tubers revealed vast changes in carbohydrate metabolism and carbohydrate content. Surprisingly the catecholamine level was changed as well. It has to be pointed out that in contrast to plants over-expressing tyrosine decarboxylase, those expressing human dopamine receptor are characterized by increases of all known tuber catecholamines (dopamine, norepinephrine and normetanephrine). Whereas norepinephrine content was positively correlated with soluble sugars and negatively with starch, normetanephrine was considered as the product of norepinephrine turnover. Increased catecholamine content was accompanied by an increase of their precursors, tyramine and L-DOPA, suggesting upregulation of the biosynthetic pathway, mediated by tyrosine decarboxylase and tyrosine hydroxylase, respectively. It is hard to explain how expression of a human receptor triggers a positive loop leading to enhanced catecholamine synthesis and turnover. It is interesting to compare data on tuber carbohydrate levels from plants over-expressing tyrosine decarboxylase (TD) with those expressing human dopamine receptor. In both cases starch content is strongly decreased, this decrease was larger for HD1 plants (from 40% to 80%) than for TD tubers (from 12% to 60%) although the norepinephrine content was higher in TD plants (Figure 6). The norepinephrine content in TD plants was about four folds higher than in HD1 plants.

Therefore we suggest that the exogenous receptor activates catecholamine action in potato plants. A difference in enzyme activities involved in starch biosynthesis was noted. The sucrose level was comparable in HD1 and TD plants and consistent with enhanced activity of SPS. Activity of starch phosphorylase was significantly increased in both TD and HD1 plants but the decreases in activity for AGPase, SuSy and PGM was seen only for HD1 plants.

### Expression of HD1 in potato (*Solanum tuberosum*) results in altered carbon metabolism

The previously reported positive correlation between catecholamine level and soluble sugars content and negative correlation with starch level for tubers of potatoes over -expressing tyrosine decarboxylase and in tubers stored at 4°C [2,7], was also found in our study. Expression of a dopamine receptor resulted in increased catecholamine content and was accompanied by decreased starch level and increases of glucose, fructose and sucrose content. It seems likely that the introduced dopamine receptor activates catecholamine action in carbohydrate metabolism. The question now arises whether catecholamine activates starch breakdown or inhibits its synthesis or whether both processes are affected.

In mammalian systems epinephrine and norepinephrine regulate glycogen turnover by stimulating glycogen mobilization and inhibiting glycogen synthesis. This appears similar in potato, with decreased starch content in HD1 tubers being a consequence of both inhibition of starch synthesis and enhanced starch mobilization. AGPase and SuSy, two key enzymes involved in starch biosynthesis, showed 44% and 32% decreases in their activities respectively. Also the activity of PGM was significantly decreased; we have not determined the contribution of the different isoforms (cytosolic and plastidial) to the observed changes. Increased content of hexose-6-phosphates demonstrates that a direct inhibition of AGPase, rather than a substrate shortage may cause inhibition of starch synthesis. Alternatively the increased hexose phosphate levels may be due to increased starch degradation in response to elevated catecholamine levels. This is supported by the increased activity of starch phosphorylase in two of four transgenic lines. The inhibition of starch synthesis and accumulation of hexose phosphates was accompanied by an increase of sucrose synthesis. Two factors should be taken into consideration. First, that SPS is subject to allosteric activation by Glc-6-P and inhibition by Pi. Second, elevated catecholamine content led to a decrease of SPS Km suggesting increase of the enzyme catalytic activty. Sucrose phosphate synthase has many potential sites of phosphorylation and three of them were shown to influence its catalytic activity. In spinach leaf, phosphorylation of Ser 158 is responsible for enzyme downregulation in darkness, phosphorylation of Ser 229 enables binding of 14-3-3 proteins and down- regulates the enzyme whereas phosphorylation of Ser 424 under stress conditions stimulates SPS activity. There is a growing body of correlative evidence that the potato tuber SPS is regulated in an analogous manner to the leaf enzyme [20].

Since the level of 14-3-3 proteins was not changed in any of the transgenic lines (data not shown) it is thus suggested that enzyme phosphorylation targeted to the stress site is responsible for its activity enhancement in HD1 plants.

In mammals the action of epinephrine and norepinephrine is mediated by phosphorylation of enzymes involved in glycogen mobilization and synthesis. Very recent studies reported direct evidence that enzymes of starch metabolism (amylopectin synthesis) are regulated by protein phosphorylation and indicate a wider role for protein phosphorylation in the control of starch anabolism and catabolism [21]. Therefore, it is possible that catecholamine action in plants could also involve phosphorylation of enzymes involved in starch metabolism.

### Catecholamines – the new stress hormones in plants?

In mammalian systems, catecholamines serve as stress hormones increasing as a result of stress. In order to see whether or not a similar response occurs in plants, leaves of potato plants were wounded and catecholamines levels prior to and 5, 10 and 13 min after wounding were determined. Although the data varied, there was a consistent increasing trend in concentration of dopamine, norepinephrine and normetanephrine [2]. Very recently a similar increase in norepinephrine was measured in potato leaves subjected to ABA and water stress treatment. Activities of both tyrosine hydroxylase (1.5 and 1.7 fold) and tyrosine decarboxylase (2.33 and 1.2fold) were increased [22]. Under normal growth conditions the major flux in potato tuber carbon metabolism is the conversion of sucrose through hexose phosphates to starch [16]. During environmental perturbations like wounding [23,24] water stress [25], high temperature [26] and hypoxia [27,28] this balance is disturbed and, consequently, large changes in tuber metabolite levels occur. Elevated temperature or water stress leads to increased respiration, a decline in 3-phosphoglycerate (3PGA), inhibition of AGPase and consequently an inhibition of starch synthesis. Decreased starch was accompanied by a stimulation of sucrose synthesis caused by increased hexose posphate levels and activation of SPS via protein phosphorylation. The activity of SuSy was decreased whereas starch mobilization was suggested to increase. These changes in carbohydrate metabolism and carbohydrate content are very similar to those observed in HD1 plants, making it conceivable that catecholamines might play a role in plant stress responses by modulating tuber primary carbon metabolism.

## Conclusions

Introducing humane dopamine receptor into plant cells can be considered as controversial but the obtained data would argue for the value of our approach.

Vast changes in the activities of key enzymes mediating carbon metabolism of potato tuber (in HD plants) led to a dramatic reduction of starch but increased sucrose content. The relation between catecholamine, primary carbon metabolism and stress seems possible. We speculate that similarly to situation in animal cells expression of HD1 in potato resulted in activation of the cAMP mediated signalling pathway. This can be supported by the result obtained for potato plants expressing another isoform of human dopamine receptor, HD2. In contrast to HD1, HD2 receptor does not affect activity of adenylate cyclase in animal cells.

Similarly plants expressing HD2 showed no changes in carbohydrate metabolism (data not shown). The obvious next step would be further investigation of our plants with respect to their kinase activity as well as cAMP levels. In parallel we have made efforts to identify a plant dopamine receptor.

## Methods

### Plant material

Potato plants (*Solanum tuberosum *L. cv. Desiree) obtained from "Saatzucht Fritz Lange KG" (Bad Schwartau, Germany) were cultivated in a greenhouse in soil under 16 h light (22°C) and 8 h dark (15°C) regime. Plants were grown in individual pots and watered daily. For analysis, the leaves were harvested at noon from 30-day-old greenhouse grown plants and the tubers were harvested in September, 3 months after the transfer of the tissue culture plants to the greenhouse.

### Construction of a transgenic plant

The 1.3 kb SmaI, XbaI cDNA encoding HD1 from Homo sapiens ((kindly provided by Marc G.Caron (Duke University, Medical Center); [EMB: XX55760])), was ligated in the sense orientation into the same restriction site of the plant binary vector under the control of the 35S CaMV promoter and Nos terminator. The vector was introduced into the *Agrobacterium tumefaciens *strain C58C1:pGV2260 and the integrity of the plasmid was verified by restriction enzyme analysis. Young leaves of wild-type potato *S. tuberosum *L.(cv. Desiree) were transformed with *A. tumefaciens *by immersing leaf explants in bacterial suspension. *A. tumefaciens *inoculated leaf explants were subsequently transferred to callus induction medium and shoot regeneration medium. Transgenic plants were pre-selected by using PCR with the primers for the respective phosphotransferase (kanamycin resistance) gene and then selected by northern blot analysis with a HD1 specific cDNA fragment as probe.

### Northern blot analysis

Total RNA was prepared from frozen plant material using the guanidinium hydrochloride method. Following electrophoresis (1.5% (w/v) agarose, 15% formaldehyde (w/v)), RNA was transferred to nylon membranes (Hybond N, Amersham, UK). Membranes were hybridised overnight at 42°C in 250 mmol sodium phosphate buffer (pH 7.2) containing 7% (w/v) SDS, 1% (w/v) bovine serum albumin (BSA) and 1 mM EDTA. Radioactively labelled full-length cDNA was used as a hybridisation probe. Filters were washed three times in 1 × SSC containing 0.5% (w/v) SDS at 65°C (highly stringent condition) or in the same buffer but at 42°C (medium stringent condition) for 30 min.

### Western blot analysis

Proteins were extracted from frozen plant material using extraction buffer E (100 mM Hepes-NaOH, pH 7.4, 10 mM MgCl_2_, 1 mM EDTA, 1 mM EGTA, 20%glycerol (v/v), 0.5 mM PMSF, 70 mM beta-mercaptoethanol) supplemented either with 0.1% or 1% TritonX- 100 (v/v). The assessment of the expression of HD1 gene by means of western blot analysis using rabbit IgG anti HD1 protein was conducted as described previously. Briefly, solubilised protein was run on 12% SDS polyacrylamide gels (w/v) and blotted electrophoretically onto nitrocellulose membranes (Schleicher and Schuell). Following transfer, the membrane was sequentially incubated with blocking buffer (5% (w/v) dry milk), and then with antibody directed against the HD1 protein (1:2000 dilution). Formation and detection of immune complexes were performed as previously described [28]. Alkaline phosphatase-conjugated goat ant rabbit IgG at a dilution of 1:1500 was used as a secondary antibody.

### Determination of starch and soluble sugar contents

Potato tuber slices and leaf discs were extracted with 80% ethanol-50 mM HEPES KOH, pH 7.4, at 80°C. The supernatant was used for enzymatic analysis of glucose, fructose and sucrose [29]. For starch measurement, extracted plant material was homogenized in 0.2N KOH, and following incubation at 95°C was adjusted to pH 5.5 with 1N acetic acid. Starch was hydrolyzed with amyloglucosidase, and the released glucose was determined enzymatically.

### Tissue extraction for catecholamine content measurement

Frozen plant tissue (400 mg) was powdered in liquid nitrogen and extracted with methanol (4 ml per g^-1 ^fresh weight), heated for 15 min at 70°C, and centrifuged (5 min., 12000 g). Samples were diluted with water to 50% methanol concentration and extracted with chloroform (1:1 v/v). A portion of the water phase was dried under vacuum and used for derivatisation [2]. Ribitol was used as an internal standard added directly to the sample homogenate (30 μg g^-1 ^fresh weight).

### GC – MS analysis

The dried extracts were incubated in pyridine/methoxyamine (20 mg mL^-1^) at 37°C for 90 min and then acidic protons were derivatised with N-Methyl- N-trimethylsilyltrifluoroacetamide (MSTFA) at 37°C for 90 min. 2 μl of sample was used for analysis [2]. A HP quadrupole mass spectrophotometer (HP 5972A), combined with a gas chromatograph HP 6890 and autosampler (all Hewlett Packard, Germany) and equipped with 30 m HP – 5MS, fused silica capillary column, was used. Injection temperature was 230°C, with the interface set to 250°C and the ion source adjusted to 180°C. The carrier gas used was helium set at a constant flow rate of 1 ml min^-1^. The temperature program was 5 min isothermal heating at 70°C, followed by a 5°C min^-1 ^oven temperature ramp to 310°C and final 1 min heating at 310°C. The system was then temperature equilibrated for 6 min at 70°C prior to injection of the next sample. Mass spectra were recorded at 2 scan s^-1 ^with an m/z 50 – 600 scanning range. The chromatograms and mass spectra were evaluated using the MSD ChemStation program (Hewlett Packard, Germany). As standards, dopamine, norepinephrine, normetanephrine, L-Dopa, tyramine and tyrosine (Sigma) were used. The recovery samples were spiked with dopamine, norepinephrine, normetanephrine, L-Dopa, tyramine and tyrosine; the estimated recoveries were 80, 93, 85, 95, 82, 90%, respectively. The following ions were used for quantification: ribitol 307; 319, L-Dopa 218; 267; 368, dopamine 174; 338; 426, norepinephrine 174; 355, 514, normetanephrine 174; 297; 456, tyramine 174; 264; 338, tyrosine 218; 280, 354. The amounts of catecholamines were determined from the ratio of peak areas of catecholamines to peak area of the internal standard (ribitol).

### Preparation and analysis of samples for enzyme activities

Tissue was harvested, weighed and immediately frozen in liquid N_2_. 0.5 g ± 0.1 g tissue was homogenised in a chilled mortar in 2 ml of an extraction buffer containing 30 mM HEPES-NaOH, pH 6.9, 10 mM DTT, 1 mM MgSO_4_, 0.5 mM EDTA, 0.5% (w/v) BSA and 0.5% (w/v) PVP at 4°C. The homogenate was centrifuged at 16000 g for 10 min and the supernatant desalted using Sephadex G-25 columns. Enzyme activities were determined using the following published methods; SPS [30]; AGPase [31]; SuSy, PK [32]; PGM [33]; starch synthase [34]; PGI, enolase [35]; UGPase [36]; hexokinase [37] and starch phosphorylase as described by [38].

### 3.9. Statistical analysis

The t-tests were performed using the algorithm embedded into Microsoft Excel. The term significant is used when P < 0.05.

## Authors' contributions

AS carried out the metabolic analysis of transgenic plants and drafted the manuscript. AŚ carried out the construction and selection of transgenic plants and performed the statistical analysis. JS conceived of the study, and participated in its design and coordination. All authors read and approved the final manuscript.

## Figures and Tables

**Table 1:** Phenotype analysis of HD1 plants revealed significant changes in single tuber mass and tuber number, whereas tubers yield was only slightly changed. Data represent the mean of determination on six individual plants per line. *Asterisks *(*) indicate values that are significantly different from the wild type plants.

	Mean FW per tuber	Tuber number per plant	Tuber weight per plant
DESI	69.49 ± 5.5	7.33 ± 1.4	509.3 ± 70.3
HD1.10	50.38 ± 3.0*	10.00 ± 1.2*	503.8 ± 42.7
HD1.27	53.39 ± 18.4	12.66 ± 5.2	675.9 ± 254.6
HD1.35	31.43 ± 10.6*	13.66 ± 3.4*	429.3 ± 125.8
HD1.36	49.73 ± 4.0*	10.50 ± 0.9*	522.1 ± 43.2

**Table 2:** Metabolite analysis of HD1 plants revealed changes in glucose-6-phosphate, fructose-6-phophate and intermediates of TCA cycle. Data represent the mean ± SE of determination on three individual plants per line. *Asterisks *(*) indicate values that are significantly different from the wild type plants.

	DESI	HD1.27	HD1.35	HD1.36
**Phosphate hexose**				

Glu-6-phosphate	4.823 ± 0,52	4.025 ± 0.36	5.605* ± 0.24	10.268* ± 0.79
Fru-6-phosphate	1,615 ± 0,14	1.301 ± 0.29	2.182* ± 0.27	4.264* ± 0.62

**TCA cycle metabolites**				

isocitric acid	21.048 ± 3.09	16.463* ± 1.42	16.295* ± 1.27	20.606 ± 2.05
citric acid	375.434 ± 27.29	118.433* ± 16.63	153.121* ± 19.25	189.106* ± 16.73
fumaric acid	0.744 ± 0.067	2.611* ± 0.124	1.400* ± 0.137	2.653* ± 0.128
malate	76.478 ± 6.29	27.878* ± 3.45	65.754* ± 4.21	63.814* ± 3.56
